# Skeletal Muscle Aging: Enhancing Skeletal Muscle Integrity and Function as a Potential Pharmacological Approach

**DOI:** 10.3390/ph18091407

**Published:** 2025-09-18

**Authors:** Sibhghatulla Shaikh, Khurshid Ahmad, Jeong Ho Lim, Syed Sayeed Ahmad, Eun Ju Lee, Inho Choi

**Affiliations:** 1 Department of Medical Biotechnology, Yeungnam University, Gyeongsan 38541, Republic of Korea; sibhghat.88@gmail.com (S.S.); lim2249@naver.com (J.H.L.); sayeedahmad4@gmail.com (S.S.A.); gorapadoc0315@hanmail.net (E.J.L.); 2 Research Institute of Cell Culture, Yeungnam University, Gyeongsan 38541, Republic of Korea; 3 Department of Health Informatics, College of Applied Medical Sciences, Qassim University, Buraydah 51452, Saudi Arabia; k.ahmad@qu.edu.sa

**Keywords:** skeletal muscle, aging, aging factors, natural compounds, pharmacological activity

## Abstract

With the increase in global life expectancy, preserving skeletal muscle (SM) health is essential for the overall well-being of older adults. Gradual decline in muscle mass, strength, and physical performance significantly contributes to frailty, reduced mobility, and heightened vulnerability to chronic diseases. These challenges underscore the need to formulate innovative strategies for safeguarding muscle health in aging demographics. This review analyzes the major biological and lifestyle factors influencing age-related changes, establishing a framework for understanding SM deterioration. The review focuses on structural and functional alterations of SM extracellular matrix components to identify potential intervention points for muscle waste mitigation. Additionally, we discuss novel interventions designed to preserve muscle mass and functionality. In older individuals, emerging pharmacological strategies, including innovative peptides and natural compounds, may mitigate catabolic processes, enhance muscle regeneration, and increase resilience. Concurrent lifestyle interventions, including specialized exercise regimens, nutritional enhancement, and stress reduction, augment these pharmacological approaches. This review provides a thorough resource for researchers, clinicians, and healthcare professionals focused on functional capacity enhancement in older adults.

## 1. Introduction

Skeletal muscle (SM), which accounts for 40% of body mass, is required for respiration, movement, metabolism, physical activity, protection, and posture maintenance [1,2]. SM is primarily made of multinucleated myofibers surrounded by mononuclear muscle stem (satellite) cells (MSCs) responsible for muscle development and regeneration following injury, damage, or atrophy [3,4]. MSCs transform into myotubes via myogenesis, which is coordinated by myogenic regulatory factors (MRFs) like Myf5, MyoD, and myogenin (MyoG). MRFs are significant contributors to prenatal and postnatal SM development and regeneration [5]. Given its importance in muscle formation, myogenesis regulation is a key focus for combating muscle atrophy.

The metabolic activities of SM, a dynamic metabolic organ that stores, uses, and delivers large amounts of energy, degrade with age, exacerbating metabolic disease onset. SM is also critical for resilience to physical stressors, and age-related muscle loss increases the likelihood of unfavorable outcomes following medical and surgical procedures [6]. A significant portion of the global population is middle-to-older-aged. In high-income nations, the proportion of those aged ≥60 is growing faster than other age groups [7]. Cumulatively, SM aging effects impose substantial socioeconomic costs [8]. Given the increasing population aging, novel treatments for improving muscle health in older people are required.

In older individuals, physical function and performance maintenance and improvement are increasingly essential. SM aging, which is characterized by decreased muscular mass and strength, frequently results in sarcopenia [9], which, in older persons, considerably contributes to falls and fractures, the second largest cause of injury and death. SM aging is characterized by a selective reduction in myofiber number and size [10], and decreased MSC activation and proliferation in response to stimuli [11]. This decreased functional contractile tissue is associated with increased fatty and fibrotic tissue deposition. Furthermore, the steady loss of innervating motor neurons and capillary density exacerbates age-related muscle strength, power, and endurance decline [6]. Although the exact cause of this enhanced atrophy is unknown, it could be because of changes in myofiber-intrinsic gene expression, the cellular microenvironment impact, or a combination of both. The understanding of the fundamental molecular pathways underpinning aging has progressed markedly. Sarcopenia is likely caused by various aging-associated molecular and cellular damages, also known as aging hallmarks or pillars, including stem cell senescence, disrupted proteostasis, denervation, genomic instability, oxidative stress, mitochondrial dysfunction, metabolic dysregulation, and chronic inflammation [12,13,14,15,16,17].

This review begins by examining the key biological and lifestyle factors influencing aging, establishing a framework for understanding the pivotal SM roles in the aging process and overall health. We then discuss how structural and functional changes in the SM extracellular matrix (ECM) influence age-related muscle decline and identify potential therapeutic targets. Furthermore, we explore potential therapeutic targets associated with SM disorders and provide an overview of current strategies for preserving muscle function and mitigating various aspects of the aging process. By integrating these perspectives, this review highlights the importance of a multifaceted therapeutic strategy in managing SM deterioration and promoting overall health improvement during the aging process.

## 2. Biological Factors Contribute to Aging

### 2.1. Muscle Stem (Satellite) Cells (MSCs)

MSC numbers decline with age, which, in mice and humans, has been demonstrated to be fiber type-specific [18,19]. In humans, MSCs decrease in type II fibers but not type I fibers [20]. Furthermore, because of enhanced proliferation, aging reduces satellite cell self-renewal capacity, which can result in apoptosis or senescence [21]. In elderly animals, satellite cell decline may contribute to sarcopenia and impaired muscle regeneration [22]. Additionally, satellite cell loss has been linked to neuromuscular degeneration with age [23]. Notably, satellite cell aging exhibits fiber-type specificity in humans: losses are more evident in type II fibers than in type I fibers [20]. This asymmetric depletion and impaired self-renewal bias regenerative failure toward fast motor units, contributing to the preferential atrophy of type II fibers and the disproportionate decline in strength and power with advancing age [24].

### 2.2. Protein Synthesis/Degradation

Older individuals exhibit anabolic resistance, i.e., reduced protein synthesis in response to nutrients and exercise [25,26]. Research indicates that protein digestion or absorption does not differ between older and younger persons, implying that anabolic resistance is caused by an increase in the amount of protein necessary to reach a protein synthesis ‘threshold’ [27].

The effects of Akt/mTORC1 are contingent upon the context. Acute activation (e.g., post-exercise combined with protein/leucine) enhances protein synthesis and repair, while chronic or dysregulated activation in aging may inhibit autophagy, disrupt proteostasis, and lead to anabolic resistance. Therefore, sustaining periodic or acute mTORC1 activation while preserving basal autophagy is likely ideal in aged muscle [28,29].

This is corroborated by studies indicating that after protein consumption, older people have lower muscle mammalian target of rapamycin (mTOR) activation [30]. Although anabolic resistance contributes to sarcopenia onset, it does not significantly affect continuous muscle mass loss in sarcopenia [31]. Cellular function depends on appropriate protein quality control. During aging, proteasomal degradation and autophagy dysregulation in various tissues result in protein aggregate accumulation, which may contribute to age-related muscle mass loss. Impaired mitophagy (mitochondrial autophagy) disrupts muscle homeostasis, resulting in the accumulation of damaged mitochondria [32]. This dysfunction has been observed in older people [33,34], implying its role in sarcopenia-associated mitochondrial dysfunction. Studies in mice lacking Atg7 in MSCs found that deficient autophagy may be involved in sarcopenia. Atg7 deletion in MSCs lineage (Atg7^ΔPax7^ mice) lowers MSC numbers in postnatal muscle, resulting in early ageing and poor regeneration, similar to aged mice. Moreover, Atg7 deletion in adult mice MSCs (Atg7^ΔPax7ER^ mice) decreases MSC number and function, underlining the importance of basal autophagy in establishing and maintaining quiescent MSCs [35]. Mouse muscle autophagy inhibition resulted in reduced survival, muscle atrophy, weakness, neuromuscular junction degeneration, mitochondrial dysfunction, and increased oxidative stress [36,37]. Additionally, by removing damaged proteins and controlling oxidative stress signaling, autophagy promotes myocyte development and muscle function [38].

### 2.3. Neuromuscular System

**Figure 1:**
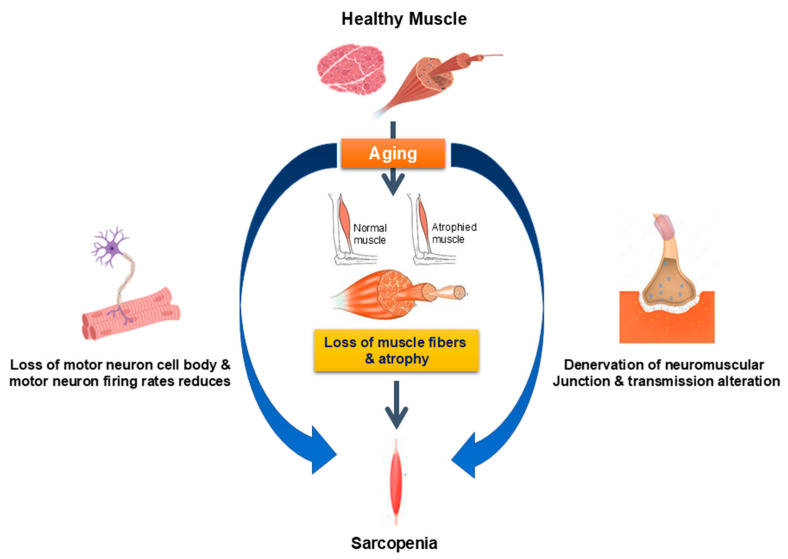
**Age-related neuromuscular deficits contributing to sarcopenia.** Motor neuron loss and decreased motor unit connectivity during aging are associated with reduced muscle size, which leads to sarcopenia.

Neuromuscular remodeling is a critical factor in muscle atrophy during aging, primarily resulting from structural and functional changes within motor units [39,40]. Aging-related neuromuscular remodeling is associated with biological changes, including impaired neuromuscular junction (NMJ) stability, decreased axonal transport efficacy, disrupted synaptic transmission, and increased oxidative stress at neuromuscular interfaces. These factors impair motor unit connectivity and integrity, resulting in reduced muscle fiber size and contractility (Figure 1) [40,41]. Denervation at the molecular level increases the expression of class IIa histone deacetylase HDAC4, which then drives the expression of the stress-response gene Gadd45a. HDAC4 and Gadd45a collaborate to regulate the activation of proteolytic pathways, specifically the ubiquitin-proteasome system and autophagy, while inhibiting Akt/mTOR-mediated anabolic signaling, which contributes to progressive muscle fiber atrophy [41,42]. These denervation–reinnervation dynamics are not evenly spread throughout the muscles; fast motor units are more likely to be affected, which speeds up the loss of type II fibers. As a result, neuromuscular remodeling increases the muscle fiber-type bias in sarcopenia, leading to greater declines in strength and power compared to endurance [43,44].

### 2.4. Antioxidant Defense Systems

Studies have found elevated oxidative stress in sarcopenia animal models [45,46]. Compared with young adults, older adults have significantly higher oxidative stress and decreased mitochondrial activity [47]. Furthermore, enhanced oxidative stress and reactive oxygen species (ROS) levels significantly increase atrogin-1 and myostatin (MSTN) expression, while suppressing myotube development [48]. In older people, MSC ROS levels are elevated, which may contribute to reduced muscle regeneration capacity [49]. Basal ROS levels also rise in aged mouse muscles [50], enhancing oxidative damage markers like protein carbonyls, lipids, DNA, and protein oxidation [51]. Although aging SM has an inherent increase in the antioxidant defense system activity [50,52], there is no additional augmentation in response to stress, such as muscle contraction, in older humans and animals [53], potentially making cells vulnerable to oxidative damage.

### 2.5. Hormones

The levels of several anabolic hormones decrease significantly with age [54]. A longitudinal observational study of 221 community-dwelling men revealed a 7% decline in plasma testosterone over four years [55], whereas daily plasma growth hormone production decreased by about 14% per decade of age [56]. Furthermore, female hormones, including estrogen, decrease during menopause [57]. In both sexes, the blood levels of insulin-like growth factor-1 (IGF-1) are significantly linked with age [58]. IGF-1 is involved in protein synthesis via the Akt/mTOR pathway [59,60], and it controls growth hormone secretion via negative feedback. Testosterone and growth hormones are strong anabolic hormones that promote muscle protein synthesis and muscle mass increase [61,62]. Estrogen may also contribute to muscle repair and regeneration by stimulating the MSCs’ proliferation [57].

### 2.6. Inflammation

Age-related inflammation, which is common in older individuals [63], is an important mechanism being studied in aging and SM health. It is characterized by increased inflammatory marker expression with age and leads to age-related sarcopenia [64,65,66]. Compared with young mice, aged mice have considerably higher proinflammatory cytokine (IL-6, TNF-α, and IL-8) plasma levels [67]. A study involving 1411 participants aged 25–91 years found that compared with younger participants, older ones had significantly higher IL-6 and TNF-α levels [68]. Elevated IL-6 levels were linked to a two-to-three-fold increase in the chance of losing >40% of muscle strength after three years of follow-up [69]. TNF-α levels also had similar associations [69,70]. Animal studies show that TNF-α causes SM protein degradation and apoptosis [71,72], potentially lowering basal muscle protein synthesis [73]. TNF-α impedes muscle regeneration by disrupting MyoD and MyoG, which are crucial transcription factors for satellite cell proliferation and differentiation [74]. Therefore, in older individuals, prolonged inflammatory cytokine elevation may inhibit satellite cell differentiation, contributing to gradual muscle loss and sarcopenia. A recent study suggests that reducing C2C12 myotube inflammation can prevent atrophic changes [75].

Sarcopenia is significantly influenced by immune cell infiltration and altered immune–muscle interaction [76]. Muscle aging results in an increase in anti-inflammatory M2a macrophages, which can lead to muscular fibrosis. Aging is associated with a shift toward chronic low-grade inflammation and reduced M2 macrophage activity, which limits MSC function and muscle regeneration [77]. In addition, T cells and other immune subsets influence muscle regeneration, and age-related dysregulation exacerbates sarcopenia [78].

### 2.7. Insulin Resistance

Aging is closely associated with type 2 diabetes (T2D), which is characterized by insulin resistance and accounts for >90% of diabetes cases [79]. T2D prevalence increases significantly from 3.1 diagnoses per 1000 individuals aged 18–44 years to 10.9 diagnoses per 1000 individuals aged 45–65 years [80]. The significant T2D onset increase during midlife is frequently linked to obesity and a lack of physical activity and coincides with SM aging onset and progression. Aging, regardless of obesity, can impair insulin sensitivity and increase the risk of T2D. In both mouse and human studies with identical body compositions, aging remained an independent driver of insulin resistance, even after controlling body fat, inflammation, and physical activity levels [81].

Insulin sensitivity, glucose regulation capacity, and muscle mass gradually decline with age [82]. Older individuals also have lower SM insulin-stimulated Akt activity [83]. Furthermore, aged mice have decreased SM insulin sensitivity, which leads to insulin resistance [84]. In an observational cross-sectional study, euglycemic–hyperinsulinemic clamp tests showed that older individuals had lower insulin sensitivity, most likely because of a higher fat mass [85]. Longitudinal studies indicate that insulin-resistant individuals lose muscle mass faster than non-insulin-resistant individuals, which increases their risk of sarcopenia [86,87]. For instance, every year, non-diabetic older individuals lose about 193 ± 22 g of muscle mass, while those with diabetes lose 293 ± 72 g [86]. The structural and functional similarities between insulin and IGF-1 imply that they may play a role in anabolic mTOR pathway activation while suppressing the catabolic ubiquitin–proteasome pathway, thereby maintaining a positive muscle protein balance [88,89]. By inhibiting these processes, insulin resistance leads to a lower net protein balance.

### 2.8. Mitochondrial Dysfunction

Mitochondrial activity is altered in aging SM, resulting in decreased energy production and enhanced oxidative stress [90]. Because they produce ATP for muscle contraction, regulate redox balance, and govern cell quality, mitochondria are crucial for muscle cell viability. Mitochondrial changes are a major sarcopenic process driver. Significant mitochondrial structure and function changes occur during SM aging. Mitochondrial fusion and fission are morphologically imbalanced, resulting in larger, rounder mitochondria, with a lower density and number, as well as matrix vacuolation and shorter cristae [91]. Aging SM has decreased mitochondrial synthesis and oxidative ability, increased ROS generation, and decreased antioxidant capacity and autophagy [92]. Mitochondrial dysfunction causes the following major age-related changes: reduced ATP synthesis [93], increased vulnerability to apoptosis [94], and increased ROS formation [95]. Overall, these findings indicate that mitochondrial dysfunction occurs with SM aging. Studies show that mitochondrial function regulation is a promising approach for SM atrophy prevention, with potential targets for the development of new treatments [96,97,98]. Fast-twitch (type II) fibers have lower mitochondrial content and oxidative capacity than slow oxidative fibers, making them more vulnerable to age-related redox stress and bioenergetic deficiencies. In humans and animal models, aging leads to a selective atrophy and loss of type II fibers, while type I fibers are relatively preserved. This effect corresponds with the greater decline in maximal strength and power compared to endurance capacity [99,100].

### 2.9. Other Biological Factors

Studies show that in older women, age-related genetic factors account for 33–50% of physical performance variation [101]. Vitamin D production decreases with age, which correlates with decreasing muscle mass and impaired physical performance [102,103]. In older individuals, elevated MSTN protein and mRNA levels are linked to reduced fat-free mass [104], implying that MSTN plays a role in sarcopenia development [105]. While MSTN expression increases with age, a recent study found that MSTN mRNA is uniquely elevated in older people with excess adiposity and insulin resistance [106]. MSTN inhibition is promising as a sarcopenia treatment, with the potential to improve SM performance [107,108].

Irisin, an exercise-induced myokine that lowers oxidative stress, has anti-inflammatory characteristics and decreases with age [109,110]. It promotes muscular growth and reduces denervation-induced atrophy in an autocrine manner [111], and its lower levels act as a muscle weakness biomarker. In animal models, irisin promotes satellite cell activation, inhibits protein degradation, and prevents necrosis, potentially relieving sarcopenia [112,113]. Continuous irisin injection in aged C57BL/6J mice reduced MuRF1, atrogin-1, and MSTN expression, which improved brown adipose function [110]. Furthermore, through the PI3K/Akt pathway, irisin reduces D-galactose-stimulated muscle fibrosis [114], indicating its potential as an age-related skeletal muscle fibrosis treatment.

Sarcopenia is caused by malnutrition, chronic inflammation, and muscle–gut axis imbalance. Studies have linked dysbiosis and sarcopenia, which are caused by oxidative stress and inflammation. Free radicals and inflammatory agents disrupt the gut mucosal barrier, allowing bacterial invasion and causing dysbiosis. Reduced gut microbiota quantity and variety enhances intestinal permeability, which raises muscle MSTN levels via the gut–muscle axis, leading to sarcopenia [115,116,117]. Certain minerals are essential for sustaining proper muscle function and metabolism. Research on community-dwelling older individuals found that compared with the highest calcium intake tertile, those with the lowest calcium consumption had a three-to-fourfold higher risk of sarcopenia [118], as well as a significantly slower gait speed [119].

Age-related muscle mass loss is caused by a complex interplay of factors that result in motor neuron degeneration and subsequent motor unit atrophy. In particular, motor end plate degeneration produces slower nerve transmission and denervation, which, combined with muscle cell atrophy, has a lifelong effect on striated muscle tissue [120]. However, these processes are reversed by the therapeutic effects of exercise. In elderly rats, endurance exercise over four months has been shown to considerably decrease nuclear pore complex protein loss while maintaining neuromuscular junction integrity and delaying motoneuron atrophy [121].

**Figure 2:**
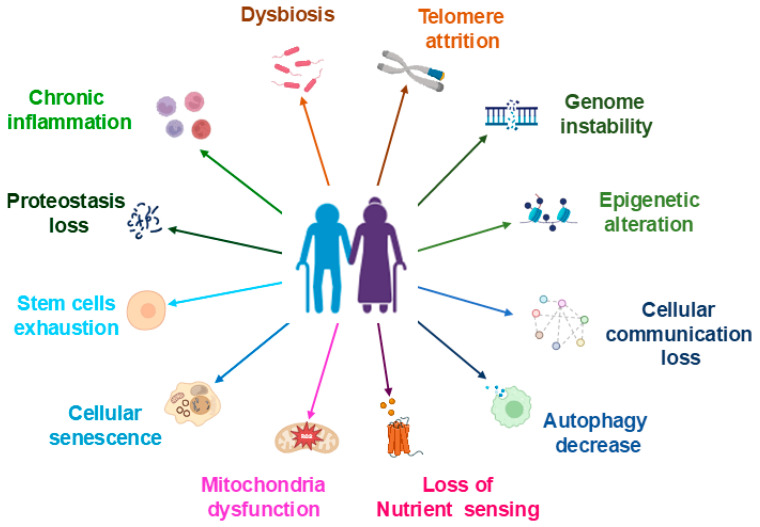
Different factors, such as dysbiosis, inflammation, epigenetic alteration, decreased autophagy, cellular senescence, and mitochondrial dysfunction, are associated with aging.

Studies indicate that individuals with lower physical activity levels are likely to develop sarcopenia [122]. In adults and older individuals, exercise and physical activity are thought to be beneficial, mitigating the adverse effects of muscle loss caused by unloading and bed rest [123,124]. Figure 2 shows the various factors associated with aging.

## 3. Skeletal Muscle ECM Dynamics in Aging

### 3.1. The ECM

In multicellular organisms, the ECM, an intricate meshwork of structural proteins, glycoproteins, proteoglycans, and polysaccharides, offers structural and biochemical support to cells, enabling them to organize, develop, and effectively perform their functions. In SM, the most abundant body tissue, the ECM is mainly made up of collagens, fibronectins, laminins, and proteoglycans. This complex network nourishes muscle fibers and MSCs and assists in transferring force and maintaining and repairing tissues. The aging process is associated with significant changes in the composition and structure of the SM ECM, which results in pathological remodeling, increased tissue stiffness, and decreased muscle function and regenerative capacity [125].

### 3.2. Skeletal Muscle ECM

The SM ECM is made up of the epimysium, perimysium, and endomysium layers. These layers are primarily made of collagen I and III, which are essential in maintaining muscle tissue structural stability and support. The epimysium is a dense connective tissue surrounding the entire muscle, while the perimysium encloses muscle fiber bundles that arise from the epimysium. The endomysium, the innermost layer, closely interacts with the myofiber sarcolemma at the basal lamina [126], a vital SM ECM layer primarily composed of laminins, collagen IV, nidogen, and perlecan [127,128]. Laminins, the main cell-adhesive components of the basal lamina, play an essential role in forming a self-assembling polymeric scaffold that supports the structure. Along with laminins, a complex interconnected network of collagen IV binds to proteoglycans, improving matrix integrity and support. This composition is essential for muscle fiber structural support, facilitating cellular attachment, and preserving the precise architecture required for muscle function and repair [128,129].

Aging causes considerable changes to the SM ECM, which is predominantly regulated by matrix metalloproteinases (MMPs) [130]. Age-related dysregulation of ECM remodeling alters the composition of the ECM, characterized by increased cross-linking and deposition of fibrillar collagen (types I and III) [131]. These factors work synergistically to induce tissue stiffness and promote fibrosis [132]. The structural changes significantly impact the behavior of muscle stem cells, impairing their ability to activate, proliferate, and migrate, resulting in cellular senescence and a diminished capacity for regeneration [133]. The ECM is a vital therapeutic target for maintaining muscular performance in aging populations, as age-related remodeling of the ECM severely undermines SM integrity and regeneration, thereby exacerbating sarcopenia.

We recently investigated several ECM components, including fibromodulin [134,135,136], matrix Gla protein (MGP) [137], fibronectin [138], and dermatopontin [139], which have been linked to myogenic regulation and SM regeneration. Fibromodulin is vital in ECM preservation and enables SM regeneration by increasing MSC recruitment to the injury site [134,135]. Dermatopontin affects myogenesis by promoting cell adhesion and myoblast differentiation, while simultaneously inhibiting cell proliferation [139]. Moreover, in MGP knockdown cells, we observed a decrease in myogenic markers and ECM gene expression, which highlights MGP’s crucial role in myogenesis regulation [137]. We have reviewed these in our recent review article [140]. Additionally, we have recently reported fibronectin-derived short peptides that significantly enhance primary and MSCs adhesion, proliferation, and differentiation, and maintain MSC viability in vitro, highlighting their potential in regenerative applications [138].

**Figure 3:**
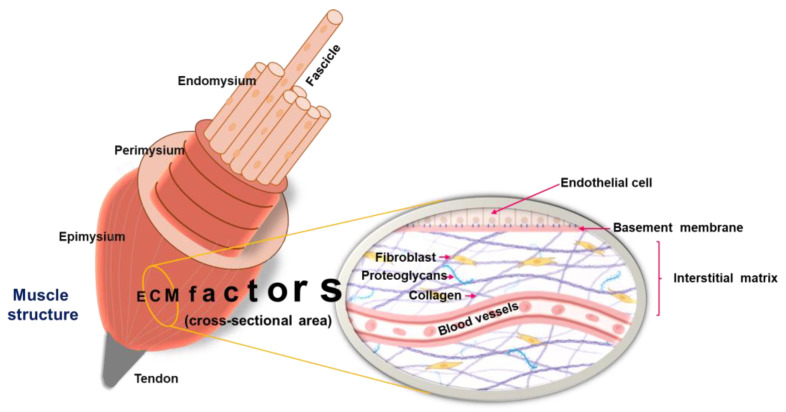
Typical structure of skeletal muscle, as well as the critical components like collagen, fibroblast, and proteoglycans associated with the ECM. Three distinct layers of SM are the epimysium, the perimysium, and the endomysium.

In response to injury or illness, the ECM undergoes considerable alterations, which influence muscle tissue function and have clinical implications [141]. ECM enhances biochemical communication, modulates cellular processes, and aids SM regeneration. By directing cell division, mobility, and specialization via cell-surface receptor contacts, the ECM regulates muscle contraction, growth, and development. It also reinforces the SM structure by connecting throughout its width. ECM also stores growth factors and aids in transmitting biochemicals and cell surface receptors. These functions are critical for muscle maintenance and recovery [141,142,143,144]. Figure 3 depicts the SM structure and ECM-associated components.

### 3.3. Effect of Aging on Skeletal Muscle ECM

Aging impacts MSCs and the ECM through various structural, metabolic, cellular, and functional changes, which contribute to a decline in muscle mechanical characteristics [145]. Aging induces several significant alterations in MSCs’ ECM composition and impacts muscle tissue structural integrity and functional capacity, contributing to common aging symptoms like decreased extensibility, increased stiffness, and impaired muscle function.

As an individual ages, collagen cross-linking increases due to both enzymatic and non-enzymatic mechanisms, including the formation of advanced glycation end products (AGEs). The cross-links modify the structural configuration of collagen fibers, leading to enhanced ECM rigidity, reduced tissue elasticity, and ultimately, age-associated SM dysfunction [146]. The levels of elastin, another key ECM protein, also change because of increased zinc-dependent proteases in older individuals, leading to ECM protein component degradation and stimulation of elastic and collagen fiber renewal [147]. Furthermore, pathological ECM deposition elevation, covalent cross-linking, and ECM remodeling dramatically increase ECM stiffness, creating a mechanical gradient between pathological and normal tissue.

Collagen content, particularly types I, III, and IV, increases with age [148]. Collagen’s increase and decrease in turnover lead to muscle tissue stiffening. Cell signaling changes because of altered ECM interactions can affect MSCs’ response to mechanical stress and repair mechanisms. ECM structural and biochemical changes contribute to a decrease in muscle strength and elasticity, resulting in a decline in overall muscle function. In aged muscles, the ECM’s ability to transmit force is compromised, which affects muscle mechanical properties and increases susceptibility to damage [149].

## 4. Therapeutic Targets and Strategies for Managing Age-Related Skeletal Muscle Decline

In older populations, therapeutic interventions for SM disorder aim to target various molecular pathways that control muscle mass, strength, and function. MSTN, a TGF-β superfamily member, inhibits muscle growth. Inhibiting MSTN activity with antibodies or small-molecule inhibitors can reduce muscle wasting and increase muscle regeneration [150]. Despite sarcopenia’s widespread occurrence and severity, there are currently no approved pharmacological treatments. Although several therapies aiming to increase protein synthesis, such as testosterone and growth hormone, have been studied, they have had little impact on muscle mass, strength, and physical function [151,152], and concerns about their safety remain [153]. MSTN signaling, a crucial component in muscle degeneration, has sparked substantial interest as a potential therapeutic target [105]. Clinical trials targeting MSTN in older adults have yielded promising results regarding muscle mass, strength, and functional outcomes [154,155,156,157]. GDF11 and activin A are promising therapeutic targets for sarcopenia due to their regulatory effects on SM function, specifically in modulating muscle mass, regeneration, and fibrosis pathways [158,159,160]. While inhibiting or genetically deleting MSTN may increase skeletal muscle mass, it has complex and adverse effects on the heart. MSTN deletion in adult mouse cardiomyocytes causes cardiac hypertrophy and dysfunction [161]. Adult male and female MSTN-null mice have larger hearts than wild-type mice, indicating that MSTN deficiency results in eccentric cardiac hypertrophy [162]. These findings imply that long-term MSTN suppression could have a negative impact on cardiac structure or function.

Several therapeutic treatments have been discovered through clinical trials, including neutralizing antibodies (Stamulumab, Domagrozumab), follistatin therapies (FS344, AAV1-FS344), soluble ACVRIIA/ACVRIIB receptors (ACE-031, ACE-2494), small-molecule inhibitors (Ly3022855, SB-431542), and MSTN propeptides. These drugs also affect TGF-β superfamily members, including bone morphogenetic proteins, GDF11, and activins. Inhibiting these factors promotes muscle mass growth but can have serious side effects on other tissues [163,164].

Addressing age-related SM deterioration requires a multifaceted approach that includes novel pharmacological treatments, targeted exercise programs, and dietary changes. The complexity of sarcopenia, which is frequently linked to diseases such as diabetes, obesity, and cancer, suggests that integrated therapies may produce better clinical outcomes. Our research has focused on identifying novel inhibitory peptides and small molecules, particularly natural compounds with therapeutic potential for diabetes, obesity, cancer, and aging-related SM mass loss [165,166,167,168,169,170,171,172,173,174,175]. The following sections provide an in-depth analysis of therapeutic agents, including proteins, peptides, amino acids, and bioactive natural compounds such as phytochemicals and vitamins, which influence critical therapeutic targets related to myogenesis and SM regeneration, aiming to maintain muscle function and improve the quality of life in the elderly. Interventions must synchronize timed mTORC1 activation with the preservation of autophagy (e.g., through endurance/AMPK-sirt1 engagement) to prevent prolonged mTORC1 hyperactivation, which may disrupt proteostasis in aging muscle [176].

## 5. Protein, Peptides, and Amino Acids as Therapeutic Agents

MSTN-derived peptides (MIF1 and MIF2) and isoleucine increased myogenesis by boosting the levels of myosin heavy chain (MyH), MyoD, and MyoG [177,178], while podocan (a short leucine-rich repeat protein) enhances C2C12 myogenic development by activating the Wnt/β-catenin signaling pathways [179]. In addition, MIF1 and MIF2 improved muscle regeneration in cardiotoxin-induced mouse muscle injury, while isoleucine increased muscle mass by promoting myogenesis and intramyocellular lipid accumulation in high-fat diet-fed C57BL/6J mice [177,178]. In burn-injured animals, insulin treatment stimulates muscle regeneration by enhancing MyoD and MyoG levels, while reducing atrophy by regulating MuRF1 [180].

Potato peptide hydrolysate PPH902 and potato alcalase hydrolysate’s decapeptide DI-10 stimulate the Akt/mTOR signaling pathway in C2C12 myoblast cells and inhibit muscle protein degradation, thereby enhancing SM development [181,182], while arginine promotes the mTOR pathway in mouse SM and increases the expression of slow-fiber genes (MyH I and Tnnt1) and mitochondrial genes (PGC-1α, TFAM, MEF2C, and NRF1) in C2C12 cells [183].

Recombinant human thioredoxin lowers oxidative stress and enhances myogenic differentiation in rat bone marrow mesenchymal stem cells [184], while Selenoprotein K (a small membrane-associated protein) regulates the endoplasmic reticulum and oxidative stress in C2C12 myoblast cells [185]. Table 1 lists SM-enhancing proteins, peptides, and amino acids.

## 6. Natural Products and Bioactive Compounds as Therapeutic Agents

### 6.1. Natural Product Extract

*Glycyrrhiza uralensis*, green tea, rosemary, *Aster glehni*, and *Saururus chinensis* extracts promote myogenesis by enhancing the MRFs (MyoG, Myf5, MyH, and MyoD) in C2C12 myoblasts [188,189,190,191,192,193]. Vigeo (a nuruk-fermented extract derived from *Eleutherococcus senticosus*, *Achyranthes japonica*, and *Atractylodes japonica*) and *Tinospora cordifolia* extract inhibit muscle protein degradation via atrogin-1 and MuRF-1 and promote C2C12 myoblast differentiation by increasing myotube width, length, and fusion [194,195]. Further, *Gloiopeltis tenax* aqueous extract enhances myogenic differentiation by increasing PGC-1α expression and mitochondrial content in C2C12 myoblasts [196].

*Luffa cylindrica Roemer* increases myotube number and diameter, and reduces atrogin-1 expression in primary rat skeletal myotubes [197], while Sea buckthorn oil has a pro-proliferative effect, increasing PCNA and Cyclin D1/CDK4 levels, and promotes myotube development by increasing MyoG expression in primary sheep myoblasts [198].

In mice, combined Korean mistletoe and apple peel extracts improve muscular strength and endurance by increasing muscle fiber size and muscle protein synthesis, reducing muscle degradation, and improving mitochondrial function [199], while Korean red ginseng (RG) stimulates SM growth by increasing tibialis anterior muscle fiber volume and mitigating injury-induced histological damage [200]. In addition, in mice with immobilization-induced muscular atrophy, fermented RG extract enhances grip strength, muscle mass, and the cross-sectional area of the gastrocnemius, quadriceps, and soleus muscles. Fermented RG extract activates the IGF-1/Protein kinase B/mTOR and Sirtuin 1 (SIRT1)/PGC-1α pathways, increasing muscle energy metabolism and inhibiting protein degradation by suppressing the FOXO3a, MuRF1, and Fbx32 pathways [201].

In aged mice, *Withania somnifera* extract increases muscle mass, prevents inflammation-associated muscular atrophy, modulates muscle protein turnover, and promotes mitochondrial biogenesis via the IGF-1/Akt/mTOR pathway [202], while in a mouse model of sarcopenia, steamed ginseng berry powder increases myotube diameter and decreases the mRNA levels of sarcopenia-associated markers [203]. Further, in CT26 tumor-bearing BALB/c mice, ajoene extract reduces muscle degradation by suppressing myokine secretion and the JAK/STAT3 and SMAD/FoxO pathways, resulting in muscle-specific E3 ligase inhibition [204].

### 6.2. Bioactive Compounds

#### 6.2.1. Licochalcone A and B

Licochalcone A and B promote myoblast proliferation and differentiation by inhibiting MSTN and decreasing atrophy-related gene (Atrogin1 and MuRF1) expression. They also exhibit anti-aging properties, indicating that they could be used as natural MSTN inhibitors for muscle loss-related condition treatment [205].

#### 6.2.2. Epicatechin

(-)-Epicatechin gallate (ECG) and (-)-epigallocatechin-3-gallate (EGCG) activate MSCs by stimulating the transcription factor, Myf5. In C2C12 myoblast cells, ECG enhances myogenic differentiation by inducing MyoG and muscle creatine kinase. Additionally, in C2C12 cells, ECG increases myotube length, diameter, and number by upregulating MyoD, MyoG, and MyH [206]. In TNF-α-treated C2C12 cells, EGCG improves protein synthesis and decreases protein degradation. In starved C2C12 myotubes, EGCG boosts protein synthesis and reduces protein degradation by inhibiting MuRF1 and atrogin-1 [207]. In mice, treatment with EGCG greatly improves muscle fiber regeneration [208]. In C2C12 myotubes, EGCG and epicatechin markedly enhance mitochondrial DNA gene expression and the activity of mitochondrial enzymes, such as citrate synthase and cytochrome C oxidase [209]. Another study found that in clinorotated C2C12 myotubes, epicatechin has an anti-atrophic effect on SM, resulting in reduced atrogin-1 and MuRF1 expression via ERK dephosphorylation [210]. In C2C12 cells and MyoD-transfected 10T/C embryonic fibroblasts, epicatechin promotes myogenesis by increasing myogenic marker expression, such as MyoD, MyoG, and MyH [211]. These results indicate that epicatechin has therapeutic potential in degenerative muscle diseases and age-related muscle weakness.

#### 6.2.3. Curcumin

Curcumin promotes SM repair and regeneration by stimulating the Wnt5a/Ca^2+^/CaN/NFAT2 signaling pathway. By upregulating Wnt5a, it stimulates MRF (MyoD, MYOG, Myf5, and MyH) expression, facilitating myogenic differentiation in C2C12 cells, as well as injured SM repair and regeneration in mice [212].

#### 6.2.4. Succinic Acid

Succinic acid enhances myogenic differentiation by increasing MyoD and MyoG expression. The succinate receptor, SUCNR1, was found in C2C12 cells, and its levels decreased during myogenesis. Despite treatment with succinic acid, intracellular succinate levels were steady but declined during myogenic differentiation. Pertussis toxin, a Gαi protein inhibitor, confirmed that succinic acid increases myogenesis via SUCNR1-Gαi signaling in C2C12 cells [213].

#### 6.2.5. Echinacoside (ECH)

ECH, a phenylpropanoid caffeic acid glycoside, promotes myogenic differentiation, glucose and fatty acid uptake, lipid catabolism, and ATP-dependent thermogenesis in vitro and in vivo. ECH stimulates mitochondrial biogenesis and thermogenic proteins while improving intracellular Ca^2+^ signaling. In C2C12 cells, it promotes myogenesis via the dopaminergic receptor 1/5 pathway and stimulates α1-AR-mediated ATP-dependent thermogenesis through the dopaminergic receptor 1/5/cAMP/SLN/SERCA1 pathway [214]. These findings highlight ECH as a potential therapeutic option for muscle-related diseases.

#### 6.2.6. 8-Prenylnaringenin

In mice, after disuse muscle atrophy, 8-prenylnaringenin improves muscle recovery by increasing Akt, 4E-BP1, and ribosomal protein S6 kinase beta-1 (P70S6K1) phosphorylation during reloading. In C2C12 cells, it also promotes PI3K, Akt, and P70S6K1 phosphorylation [215]. A diet with 8-prenylnaringenin enhanced gastrocnemius muscle growth by activating Akt and suppressing atrogin-1 [216].

#### 6.2.7. Dieckol and 2,7-phloroglucinol-6,6′-bieckol

Marine algal polyphenols, such as dieckol and 2,7-phloroglucinol-6,6′-bieckol, promote C2C12 myoblast proliferation and regulate myogenesis by inhibiting Smad signaling and increasing IGF-1 signaling, which significantly increases p-Akt and p-FoxO protein levels. Docking analyses showed that these compounds have a high MSTN and IGF-1 receptor affinity [217].

#### 6.2.8. Dihydromyricetin (DM)

DM’s anti-atrophic effects have been studied in a DXM-induced muscular atrophy model. In vivo, DM maintains muscle mass, increases fiber cross-sectional area, and improves grip strength. In vitro, it enhances MyH content and myotube diameter. Furthermore, DM increases mitochondrial biogenesis, restores mitochondrial function, and enhances protein metabolism, thereby preventing DXM-induced muscle atrophy [218].

Treatment with D-galactose promotes aging by increasing oxidative stress, which impairs autophagy and causes excessive apoptosis [219]. In D-galactose-treated animals, DM reduces muscle atrophy by activating the AMPK/SIRT1/PGC-1α pathway, increasing autophagy and inhibiting apoptosis and the ubiquitin–proteasome system. It also increases the gastrocnemius muscle-to-body weight ratio [220].

#### 6.2.9. Ascorbic Acid (Vitamin C)

In cultured myoblast from patients with osteoporosis, ascorbic acid (AsA) reduces ropivacaine’s adverse effects. In SM, ropivacaine increases ROS levels and the expression of Nox4, an enzyme associated with ROS generation. Treatment with AsA reverses ropivacaine’s oxidative effects and partially restores basal conditions. Ropivacaine decreases myogenic markers like MyoD and Myf5 while increasing MSTN, and treatment with AsA reverses ropivacaine-induced dysregulation of myogenic regulators [221]. Another study found that in C2C12 cells, AsA increases the levels of sodium-dependent vitamin C transporter 2 and cysteine-rich protein 3. It also enhances C2C12 cell differentiation and muscle regeneration in injured mouse muscle by promoting cysteine-rich protein 3 nuclear translocation, which then interacts with MyoD and MyoG [222].

The functional interplay between vitamin C and capsaicin, an ER stress inducer, has been investigated in myogenesis. In undifferentiated C2C12 cells, AsA and ascorbic acid phosphate (AsAp) showed little influence on myogenesis. Capsaicin (300 μM) enhanced ER- and oxidative stress-associated gene expression. Notably, combining treatment with capsaicin one day before differentiation and treatment with AsAp four days after differentiation significantly improved myogenesis, resulting in long myotube formation and elevated MyH1/2, MyH1, MyH4, and MyH7 expression. Mild ER stress appears to enhance myogenesis, and AsAp may alleviate oxidative stress effects in capsaicin-pretreated C2C12 cells. The increased MyH1 and MyH4 expression was associated with increased Col1a1 expression, which shows that altering the myogenic microenvironment is critical for effective myogenesis [223]. These results indicate that AsA may accelerate myogenesis in a cell context-dependent manner.

#### 6.2.10. Silibinin

Exposing C2C12 myotubes to S2-013 pancreatic cell-conditioned media enhances MuRF1 and atrogin-1 expression, resulting in decreased protein content. Treatment with silibinin reverses these effects by restoring protein levels and suppressing MuRF1 and atrogin-1 expression. In mice with S2-013 tumors, silibinin decreases tumor size, reduces gastrocnemius muscle atrophy, and enhances grip strength. It also reduces the muscle levels of MuRF1, atrogin-1, IL-6, and TNF-α, suggesting that its anti-cachectic effects are linked to the downregulation of these markers [224].

#### 6.2.11. Omega-7 Palmitoleic Acids

An investigation of the effect of a hyperglycemic environment on L6 myoblast development and the role of omega-7 palmitoleic acid (PMA) revealed that exposure to high glucose (25 mM) increases MRF (MyoD, MyoG, MRF4, MyH2x, and MyH2a) expression and myosin production, as well as activates the PI3K/AKT pathway, thereby enhancing myoblast differentiation. Hyperglycemia also raised the levels of ROS and the inflammatory cytokine, Tnfaip3. PMA was found to decrease MRF expression and upregulate Pax3, a proliferation-related gene. Trans-PMA increases MAPK/ERK1/2 phosphorylation while lowering ROS and Tnfaip3 levels. In contrast, cis-PMA inhibits MAPK/ERK1/2, increasing ROS production [225]. Overall, hyperglycemic conditions favor excessive myoblast development, but this differentiation was prevented by PMA via MRF downregulation. Furthermore, trans-PMA may have antioxidant and anti-inflammatory properties in muscle cells.

#### 6.2.12. Oleic Acid

In C2C12 myotubes, oleic acid supplementation stimulates type 1 fiber production, and in mice, it improves running endurance [226,227]. It also changes muscle fiber composition by increasing type 1 and 2X fibers in the soleus and type 2X fibers in the extensor digitorum longus muscle [227].

#### 6.2.13. 5,7-Dimethoxyflavone

In 18-month-old C57BL/6J mice, 5,7-dimethoxyflavone increases exercise capacity, grip strength, and muscle mass. In the gastrocnemius muscle, it enhances mTOR activity while decreasing MuRF1 and atrogin-1-mediated proteolysis via the PI3K/Akt pathway. Additionally, 5,7-dimethoxyflavone enhances mitochondrial DNA content and improves energy metabolism by activating PGC-1α [228]. In C2C12 cells, it increases glycogen content and glycogen synthase mRNA expression [229].

#### 6.2.14. Apigenin

In C2C12 myoblasts, apigenin suppresses lipopolysaccharide-stimulated atrogin-1/MAFbx expression by inhibiting the JNK signaling pathway [230]. It also inhibits palmitic acid-stimulated muscle atrophy and mitochondrial dysfunction in C2C12 cells and reduces obesity-induced SM atrophy by increasing mitochondrial function [231]. In denervated mice, apigenin inhibits muscle atrophy by decreasing MuRF1 expression, while increasing MyH and MyH type IIb expression. Additionally, it reduces the expression of TNF-α in the gastrocnemius and soleus muscles, as well as IL-6 in the soleus muscle [232].

#### 6.2.15. Baicalin

Baicalin reduces anorexia by inhibiting cytokine expression (TNF-α and IL-6), prevents muscular atrophy by reducing MuRF1 and atrogin-1 expression, and inhibits NF-κB activation in a cancer cachexia model [233]. Cancer-related muscle wasting is associated with increased NF-κB activity and enhanced levels of circulating activin-A, a negative muscle mass regulator. A clinical trial involving cancer patients found that taking 50 mg of baicalin daily for three months enhanced lean body mass and reduced serum NF-κB and activin-A levels [234].

#### 6.2.16. Andrographolide

In a cardiotoxin-induced injury model, andrographolide enhances SM regeneration by promoting myotube formation and fusion. Treatment with andrographolide increases the expression of MRFs, such as Desmin, MyoD, MyoG, Myomaker (a fusion marker gene), Tnni2, Dmd, Myoz1, and Myoz3. Andrographolide stimulates histone modification (H3K4Me2, H3K4Me3, and H3K36Me2) in vivo and in vitro. DZNep, an inhibitor of the lysine methyltransferase EZH2, reduces andrographolide-induced Myf5, Myomaker, MyoD, MyoG, and SM α-actin expression [235]. These findings suggest that andrographolide regulates myotube formation and fusion epigenetically, making it a promising therapeutic for muscle regeneration after injury.

#### 6.2.17. Luteolin

In lipopolysaccharide-stimulated C2C12 myotubes, luteolin enhances myotube diameter by inhibiting the JNK pathway and downregulating atrogin-1 [230]. In vivo, luteolin decreases TNF-α and IL-6 levels, suppresses muscle protein breakdown genes (MuRF1 and atrogin-1), and prevents cancer-induced muscle wasting [236].

#### 6.2.18. Sinensetin

In a study of primary MSCs from thigh and calf tissues of young and old rats, sinensetin treatment increased old rats’ MSC differentiation compared to nontreated control old group cells. It also increased MyoD and MyoG protein levels in MSCs from old rats [237]. Sinensetin may help age-related sarcopenia by enhancing MSCs differentiation and protein expression.

#### 6.2.19. β-Carotene (BC)

In C2C12 myotubes and mouse soleus muscles, BC, a provitamin A carotenoid and antioxidant that effectively scavenges free radicals [238], shows protection against H_2_O_2_-induced muscular atrophy [239]. In mice, combined treatment with BC, astaxanthin, and resveratrol increases protein synthesis during muscle hypertrophy following atrophy [240]. Additionally, in a Lewis lung carcinoma (LLC)-induced cancer cachexia mouse model, BC supplementation reduces tumor development and inflammatory cytokines, while enhancing muscle weight and strength. These effects are most likely mediated by the PI3K/Akt pathway, which regulates muscle atrophy. In C2C12 myotubes, BC significantly alleviates muscular differentiation defects and atrophy induced by LLC-conditioned media [241]. These findings point to BC as a potential treatment for cancer cachexia.

#### 6.2.20. Morin

A cachexia study found that compared with controls, muscle wet weights and myofiber size were lower in LLC mice. However, these reductions were countered by morin intake. Morin-fed mice also had lower tumor weights, and treatment with morin reduced cell viability and protein synthesis in LLC cells [242].

#### 6.2.21. Quercetin

Quercetin effectively suppresses the activity of MSTN, ACVR2B, and SMAD2 and 3, and significantly enhances MSC differentiation and myotube formation in vitro [243]. In C2C12 cells, it stimulates migration and early myogenic differentiation by activating p-IGF-1R via molecular mechanisms that include upregulating the transcription factor, STAT3, and the AKT signaling pathway, and promoting myoblast migration via ITGB1 signaling, which activates FAK and phosphorylates paxillin [244]. In DXM-treated C2C12 cells, quercetin enhances cell viability and prevents apoptosis by regulating mitochondrial membrane potential and reducing oxidative species [245]. In a DXM-induced atrophy model, quercetin glycosides increase gastrocnemius muscle weight while decreasing the expression of muscle atrophy-related genes, including MSTN. In C2C12 myotubes, quercetin increases Akt phosphorylation and atrogene expression, which are important MSTN pathway components [246]. These findings highlight quercetin’s potential to promote muscle regeneration.

In myotubes, quercetin inhibits TNFα-induced expression of the atrophic factors, MAFbx/atrogin-1 and MuRF1, while increasing heme oxygenase-1 (HO-1) levels and promoting Nrf2 nuclear translocation. In obese mice fed a high-fat diet, quercetin reduces muscle atrophy, increases HO-1, and inhibits NF-κB [247].

#### 6.2.22. Genistein

Genistein affects SM growth by regulating protein degradation [248], oxidative stress [249], and myotube formation [250]. Recent in vitro and in vivo findings show that genistein improves muscle regeneration by increasing MSC differentiation in the middle to late stages of muscle injury by modulating miR-221/222 expression, indicating its potential applications in muscle regeneration [251].

#### 6.2.23. α-Mangostin

α-Mangostin increases C2C12 cell survival and promotes myogenic differentiation by upregulating MyoD and MyoG protein expression [252].

#### 6.2.24. Glabridin

In KK-Ay/Ta mice, glabridin-containing licorice flavonoid oil boosted femoral muscle growth without body weight changes by lowering p-p38 and enhancing p-mTOR protein levels [253]. In C2C12 myotubes, glabridin reduced DXM-stimulated protein degradation by downregulating the expression of the ubiquitin ligases, MuRF1 and Cbl-b. This was achieved by glabridin’s direct binding to the glucocorticoid receptor, which prevented DXM binding and inhibited the receptor’s DXM-induced nuclear translocation, as well as p38 and FoxO3a phosphorylation, which regulate ubiquitin ligases [254].

#### 6.2.25. Corylifol A

Corylifol A enhances myogenic differentiation by increasing MyoD transactivation and upregulating myogenic markers, such as MyoD, MyoG, and MyH. It promotes multinucleated, MyH-expressing myotubes and exerts its effects via the p38 MAPK pathway. By increasing multinucleated myotubes, decreasing muscle-specific ubiquitin-E3 ligases (MAFbx and MuRF1) and MSTN expression, and activating Akt, corylifol A protects against DXM-induced myotube loss [255]. Overall, corylifol A reduces muscle atrophy by increasing myoblast differentiation and preventing muscle breakdown.

#### 6.2.26. Melatonin

A study created stable Pax7-knockdown C2C12 cells and then evaluate muscle degradation and regeneration markers after melatonin treatment in growth and differentiation media. In silico RNA sequencing analysis revealed that melatonin enhances muscle development through the Wnt signaling pathway. An miRNA database screening identified that miR-3475-3p is a specific binding site on Pax7 and function as a negative Pax7 regulator, which mediates melatonin-stimulated myoblast differentiation. In a mouse model of glycerol-induced muscle damage, early treatment with melatonin improved Pax7 expression, myoblast differentiation, and fiber morphology [256]. These data imply that the melatonin/Pax7 axis might be an important therapeutic target for improved muscle recovery.

#### 6.2.27. Daidzein

Daidzein inhibits DXM-induced atrophy by activating Akt and stimulates myogenesis via the Akt/p38MAPK pathway, thereby increasing MyoD expression level, a key myogenic transcription factor. In mouse embryonic fibroblasts, Daidzein promotes myotube formation via the Akt/mTOR/S6K pathway and increases MyoD-driven myogenic conversion [257]. Treating C2C12 cells with daidzein significantly increases myotube diameter and number while upregulating MyH expression [258].

#### 6.2.28. Sulforaphane (SFN)

SFN has various effects on human health and disease. A recent study found that treating C57BL/6J mice with SFN enhances body weight and increases longissimus dorsi muscle fiber cross-sectional area and diameter. Furthermore, in the longissimus dorsi muscle, SFN lowers triglyceride and total cholesterol levels. SFN also modulates lipid and protein metabolism pathways, such as AMPK, fatty acid, cholesterol, PPAR, TGFβ, and mTOR signaling [259].

#### 6.2.29. Delphinidin

In DXM-treated C2C12 cells, delphinidin inhibits MuRF1 upregulation while preventing miR-23a and NFATc3 downregulation. In vivo, oral delphinidin administration prevents gastrocnemius muscle mass loss while suppressing MuRF1 expression and increasing miR-23a and NFATc3 levels [260]. These data indicate that delphinidin may prevent disuse muscle atrophy by regulating miR-23a and MuRF1 expression.

#### 6.2.30. Eicosapentaenoic Acid (EPA)

EPA activates phosphoglycerate mutase 2 (PGM2), which converts 2-phosphoglycerate to 3-phosphoglycerate, a critical glycerol degradation step that promotes MSC proliferation and differentiation in SM. PGM2 also suppresses mitochondrial metabolism, which promotes fast-twitch muscle fiber development. Interestingly, EPA and PGM2 knockdowns have opposing transcriptomic effects, primarily in the PI3K–AKT pathway. PGM2 regulates the PI3K/AKT signaling pathway, reducing FOXO1 phosphorylation, which reduces mitochondrial function and promotes fast-type muscle fiber development [261]. Overall, by targeting PGM2 and controlling the PI3K/AKT pathway, EPA stimulates muscle development and regulates glucose metabolism.

#### 6.2.31. Isobavachalcone (IBC)

In C2C12 myotubes, IBC stimulates MyoD transcriptional activity and MyH protein expression [262]. In TNF-α-treated C2C12 cells, IBC reduces muscle atrophy markers (MuRF1 and MAFbx) and restores MyH and MyoG expression. Furthermore, IBC regulates NF-κB and p38 phosphorylation while upregulating Nrf-2 and HO-1, which are important oxidative stress regulators [263]. Overall, IBC can help reduce muscle atrophy by targeting inflammation and oxidative stress pathways.

#### 6.2.32. Glyoxylic Acid

In C2C12 cells, glyoxylic acid increases the expression of MRFs, such as MyH II, MyoD, and MyoG, as well as mitochondrial biogenesis. In C2C12 and L6 cells, it suppresses DXM-induced activation of ubiquitin ligases (Trim63 and Fbxo32), which are critical muscle atrophy markers, which implies it has a protective effect against DXM-induced muscle atrophy [264].

#### 6.2.33. Oleocanthal

Oleocanthal, a monophenol found in extra virgin olive oil, reduces muscle wasting in TNF-α- or C26 tumor-conditioned medium-induced C2C12 myotubes. Treatment with oleocanthal restores myotube size and morphology, as well as normal levels of muscle atrophy markers (atrogin-1 and MuRF1) and MRFs (Pax7, MyoG, and MyH) [265]. These findings suggest that oleocanthal protects against muscle-wasting stimuli.

#### 6.2.34. Panduratin A

In TNF-α-treated L6 SM cells, panduratin A increases myotube diameter by activating the PI3K/Akt/mTOR pathway and upregulating MyoD and MyoG expression levels. By reducing E3 ubiquitin ligase and autophagy-related gene expression, while increasing catalase and superoxide dismutase mRNA levels, it inhibits ROS formation [266]. These findings highlight panduratin A as a potential muscular atrophy treatment.

#### 6.2.35. Sophoranone

Sophoranone increases C2C12 cell proliferation and differentiation. In a rabbit model of acute medial rectus muscle injury, sophoranone enhanced muscle regeneration by increasing creatine kinase levels and upregulating MRF (MyH3 and MyoG) expression in injured muscles [267].

#### 6.2.36. Diosmin

Diosmin promotes C2C12 myoblast proliferation by stimulating the Akt/FOXO1 pathway, which results in FOXO1 nuclear export. This mechanism, which inhibits p27 expression while increasing CDK2, CDK4, cyclin D1, and cyclin E1, accelerates cell cycle progression and promotes myogenesis [268].

#### 6.2.37. Ginsenoside Rg5

Ginsenoside Rg5 stimulates C2C12 myoblast development by increasing p38MAPK phosphorylation and MyoD/E2A heterodimerization. It also inhibits DXM-induced muscle atrophy and promotes myogenesis via Akt/mTOR activation. Furthermore, Akt activation phosphorylates FoxO3a, reducing Atrogin-1 and MuRF1 expression [269].

#### 6.2.38. Resveratrol

C2C12 cells were treated with resveratrol in the presence of various glucose concentrations, and metabolic and clock proteins were measured. Resveratrol stimulates SIRT1, AMPK, and protein phosphatase 2A, whereas higher glucose levels enhance mTOR signaling activity in myotubes. However, although resveratrol did not activate the mTOR pathway, it activated P70S6K and ribosomal protein S6. P70S6K activation, which was mTOR signaling-independent, increased MyoG expression, thereby promoting muscle growth and improving circadian rhythm [270]. A study involving obese mice found that after injury, resveratrol increases MSC proliferation and differentiation while upregulating mitochondrial biogenesis markers. In C2C12 cells, resveratrol activated AMPK and increased PGC-1α expression, which improved myogenic differentiation [271]. Furthermore, a recent study found that dietary resveratrol enhances myofiber diameter, amino acid content, antioxidant marker levels, and mTORC1 and MyH9 expression levels in Siberian sturgeon muscles [272].

#### 6.2.39. Maslinic Acid (MA)

In mouse gastrocnemius muscles, MA inhibits denervation-induced muscle atrophy and weakness. Gene set enrichment analysis in MA-treated mice identified the potential mechanisms underlying muscle preservation, including myogenesis and PI3K/AKT/mTOR, TNFα/NF-κB, and TGF-β signaling. MA-mediated IGF-1 upregulation and Atrogin-1, Murf1, and TGF-β expression suppression were confirmed using qPCR [273], which indicated that MA has the potential to prevent muscle atrophy and strength loss.

#### 6.2.40. Ferulic Acid (FA)

In adult male zebrafish, FA promotes growth and myogenic development by enhancing MRFs (MyoD, MyoG, Myf5) and increasing SM fiber width. FA increases PPAR-α, thereby promoting fat metabolism, and decreases PPAR-β and PPAR-γ levels, lowering fat deposition in zebrafish [274]. These results suggest that FA has the potential to promote SM growth.

#### 6.2.41. Theaflavin

Theaflavins, bioactive substances found in black tea, possess health benefits, including myogenesis stimulation in C2C12 cells. They promote myoblast development by controlling cell cycle withdrawal, activating MRFs (MyoD, MyoG, and MyH), and accelerating myogenic differentiation. They also modulate myoblast adhesion, migration, fusion, and the mechanical properties of mature myotubes [275]. These findings imply that for age-related muscle maintenance, theaflavins might be a useful dietary supplement.

#### 6.2.42. Paeoniflorin

Paeoniflorin prevents C26 colon cancer cell- or LLC-conditioned media-stimulated myotube atrophy by lowering Atrogin-1 expression while maintaining MyH and MyoD levels. In C26 tumor-bearing mice, paeoniflorin reduces weight loss, muscle fiber atrophy, and functional impairment. Paeoniflorin inhibits the TLR4/NF-κB pathway and activates the AKT/mTOR pathway both in C2C12 cells and tumor-bearing mice. Additionally, it reduces IL-6 production in C26 cancer cells [276]. These data highlight paeoniflorin as a viable cancer cachexia therapy. Table 2 lists SM-enhancing plant extracts and natural compounds.

## 7. Conclusions and Future Directions

A progressive decline in muscle mass and strength presents a significant challenge for aging populations, underscoring the urgent need for innovative and diverse treatment strategies. While this review focuses on pharmacological options, structured physical exercise, particularly resistance and power training combined with sufficient protein intake, remains the most effective primary intervention for age-related muscle decline. Pharmacological methods should be viewed as supplementary or alternative options only when exercise is not possible or proves inadequate. Despite notable progress in understanding SM degradation mechanisms, applying these findings to effective treatments requires further research and rigorous clinical testing. New therapies, including novel peptides, natural compounds, and multi-targeted pharmacological approaches, hold great promise to preserve muscle function and improve patient outcomes.

Looking ahead, priority biological targets include (i) modulation of the MSTN/activin A/GDF11 axis with careful cardiometabolic safety monitoring; (ii) stabilization of neuromuscular junctions (e.g., HDAC4–Gadd45a stress pathways; agrin/MuSK); (iii) restoration of mitochondrial quality control and bioenergetics (AMPK–SIRT1–PGC-1α signaling, mitophagy), with attention to preserving type II fibers; (iv) reduction of chronic low-grade inflammation (IL-6/TNF) and enhancement of insulin/IGF-1–Akt/mTOR signaling in sarcopenic phenotypes with metabolic comorbidities; and (v) strategies for ECM remodeling (balancing TGF-β/MMP activity) to improve the satellite cell niche.

To accelerate translation, we suggest exercise-first, pharmacology-adjunct care pathways; patient stratification by fiber-type composition, neuromuscular status, and metabolic comorbidities (e.g., T2D/obesity); use of bridging biomarkers (e.g., circulating myostatin/activin A, IL-6, myomiRs) linked to functional endpoints (grip strength, leg-press power, gait speed, stair-climb power, SPPB) and body composition measures (DXA lean mass); human tissue validation of mechanisms identified in animal or cell models; muscle-targeted delivery and dosing-window optimization; and pragmatic trials in multimorbid older adults to establish effectiveness, adherence, and scalability. A multidisciplinary, exercise-anchored strategy integrating these elements offers the most realistic path to preserving function, independence, and quality of life in aging populations.

## Figures and Tables

**Table 1:** Protein, peptides, and amino acid that enhanced skeletal muscle.

Protein, Peptides/Amino Acid	Concentration/Dose	Mechanism	Object/Model	Reference
MIF1 and MIF2	1000 nM	MSTN (↓), proliferation and differentiation (↑)	Cell line (mouse C2C12)	[178]
	1.125 mM	muscle regeneration	Animal (mouse, cardiotoxin-induced C57BL/6 male mice)	
Isoleucine	25 g/L	muscle and fat mass (↑)	Animal (C57BL/6J mice fed a high-fat diet)	[177]
Potato peptide hydrolysate	2.5, 5, and 10 µg/mL	Akt/mTOR signaling (↑)	Cell line (mouse C2C12)	[181]
Whey protein	100, 500, and 1000 µg/mL	IGF-1 (↑); myotube formation (↑)	Cell line (mouse C2C12)	[186]
Thioredoxin	2 µg/L	myogenic differentiation (↑)	Primary cells (rat bone marrow mesenchymal stem cells)	[184]
Selenoprotein K	-	regulates ER stress and oxidative stress	Cell line (mouse C2C12)	[185]
Galectin-1	0.054, 0.11, and 0.22 μM	MyoG (↑), MyoD (↑), and MyH (↑)	Human-Murine myoblasts H2K A/J^−/−^	[187]
Insulin	5 UI/Kg	muscle regeneration (↑)	Animal (rat, burn-injured animals)	[180]
Podocan	1.5 mg/mL	myogenic development (↑)	Cell line (mouse C2C12)	[179]
Arginine	Diet with 0.5%, or 1.0% arginine	MyH I (↑), MyH IIA (↑), PGC-1α (↑), NRF1 gene expression (↑), mTOR pathway (↑)	Animals (C57BL/6J male mice)	[183]
	1.2 mM or 3.6 mM	mitochondrial genes (PGC-1α, TFAM, MEF2C, and NRF1) (↑)	Cell line (mouse C2C12)	

Evidence source is standardized as Human, Animal (mouse/rat), Primary cells, or Cell line to clarify translational context.

**Table 2:** Plant extract and natural compounds that enhanced skeletal muscle.

Natural Resources (Compounds and Extract)	Concentration/Dose	Mechanism	Object/Model	References
*G. uralensis*	100 µg/mL	MSTN (↓), myogenesis (↑)	Cell line (mouse C2C12)	[188]
Green tea extract	10 µg/mL	MyoG (↑), Myf5 (↑), and MyoD (↑)	Cell line (mouse C2C12)	[189]
Rosemary extract	20 µg/mL	MyoG (↑), and MyoD (↑)	Cell line (mouse C2C12)	[190]
*Aster glehni* extract	-	MSTN (↓), MyoD (↑), myoglobin (↑), myosin (↑), and ATP generation (↑)	Cell line (mouse C2C12)	[191]
Gloiopeltis tenax aqueous extract	10 µg/mL	myogenic differentiation (↑); Atrogin/MuRF1 pathway (↓)	Cell line (mouse C2C12)	[196]
	20 mg/kg	mitochondrial biogenesis and function (↑)	Animal (mouse, DXM-induced atrophy)	
Hachimijiogan	0.1 mg/mL	MyoG (↑)	Cell line (mouse C2C12)	[192]
		muscle wasting (↓)	Animal (cancer-cachectic mice)	
*Tinospora cordifolia* extract	0.05 mg/mL	myogenic differentiation (↑)	Cell line (mouse C2C12)	[194]
Sea buckthorn oil	-	PCNA (↑), Cyclin D1/CDK4 (↑), and MyoG (↑)	Primary cells (sheep myoblasts)	[198]
Korean mistletoe and apple peel extracts	100 mg/kg or 200 mg/kg	muscular strength and endurance (↑)	Animal (mice)	[199]
Luffa cylindrica Roemer	200 and 400 μg/ml	myotube number and diameter (↑)	Primary cells (rat skeletal myotubes)	[197]
Saururus chinensis extract	10 ng/ml	MyH (↑)	Cell line (mouse C2C12)	[193]
Withania somnifera extract	200 mg/kg	muscle mass (↑)	Animal (aged mice)	[202]
	200 and 300 mg/kg	TNF-α and IL-1β (↓)	Animal (12-month-old mouse model)	
Korean red ginseng	100 and 400 mg/kg	SM growth (↑)	Animal (mice)	[200]
Ginseng Berry Powder	25 or 50 mg/kg	myotube diameter (↑)	Animal (mouse, sarcopenia model)	[203]
Vigeo	10, 25, and 50 µg/mL	myotube width, length, and fusion (↑); MyH (↑), MyoD (↑), and MyoG (↑)	Cell line (mouse C2C12)	[195]
Sialyllactose	100 mg/kg	exercise performance (↑)	Animal (mice)	[277]
Ajoene	5 mg/kg or 10 mg/kg	muscle degradation (↓)	Animal (mouse, BALB/c, CT26 tumor)	[204]
	100 nM	Myogenesis (↑)	Cell line (mouse C2C12)	
Licochalcone A and Licochalcone B	1 ng/mL, and 3 mg/kg	MSTN (↓); Atrogin1 (↓), and MuRF1 (↓)	Cell line (mouse C2C12) & Animal (mouse, C57BL/6)	[205]
Epicatechin	10 µM, and 20 µM	myogenic differentiation (↑)	Cell line (mouse C2C12)	[206]
Curcumin	80 mg/L	MyoD (↑), MYOG (↑), Myf5 (↑), and MyH (↑)	Cell line (mouse C2C12)	[212]
Succinic Acid	10–1000 µM	MyoD (↑), and MyoG (↑)	Cell line (mouse C2C12)	[213]
Echinacoside	1, 5, and 10 µM	Myogenesis (↑)	Cell line (mouse C2C12)	[214]
8-Prenylnaringenin	-	muscle recovery (↑)	mice	[215]
Dieckol and 2,7-phloroglucinol-6,6′-bieckol	5 nm, 10 nm, and 20 nm	Smad signaling (↓), and IGF-1 signaling (↑)	Cell line (mouse C2C12)	[217]
Dihydromyricetin	100 mg/kg, and 200 mg/kg	fiber cross-sectional area (↑)	Animal (mouse, DXM-induced atrophy)	[218]
Ascorbic acid	6 g/L through drinking water	Differentiation (↑), and muscle regeneration (↑)	Animal (injured mouse muscle)	[222]
Silibinin	200 mg/kg	grip strength (↑), MuRF1 (↓), Atrogin-1 (↓), IL-6 (↓), and TNF-α (↓)	Animal (cachectic mice)	[224]
Omega-7 palmitoleic acids	100 µM	Pax3 (↑), and ROS (↓)	Cell line (L6 myoblast)	[225]
Oleic acid	Diet supplemented with 10% (*w*/*w*) oleic acid	running endurance (↑)	Animal (mice)	[226,227]
5,7-Dimethoxyflavone	25 or 50 mg/kg	exercise capacity (↑), grip strength (↑), and muscular mass (↑)	Animal (18-month-old C57BL/6J mice)	[228]
Apigenin	0.1% apigenin-containing diet	muscle atrophy (↓); MyH and MyH type IIb (↑)	Animal (mouse, denervation model)	[232]
Baicalin	50 and 150 mg/kg	TNF-α and IL-6 (↓); MuRF1 and Atrogin-1 (↓)	Animal (mouse, cancer cachexia model)	[233]
Andrographolide	10 mg/kg	SM regeneration (↑)	Animal (mouse, cardiotoxin-induced injury model	[235]
Luteolin	20 mg/kg	TNF-α and IL-6 (↓)	Animal (cancer-induced muscle wasting in vivo)	[236]
Sinensetin	50 and 100 μM	MSC differentiation (↑)	Animal (aged rats)	[237]
Vitamins A	100 nM	muscle growth (↑)	Primary cells (bovine MSC)	[278]
β-carotene	4 or 8 mg/kg	tumor development (↓); muscle weight and strength (↑)	Animal (mouse model of cancer cachexia)	[241]
Quercetin	1, 10, and 100 nM	MSTN (↓); MSC differentiation and myotube formation (↑)	Cell line (mouse C2C12)	[243]
Genistein	10 µM, and 10 mg/kg	MSC differentiation (↑)	in vitro and in vivo	[251]
α-Mangostin	3, and 6 μM	MyoD and MyoG protein (↑)	Cell line (mouse C2C12)	[252]
Glabridin	0.1, 1, and 10 μM	DXM-stimulated protein degradation (↓)	Cell line (mouse C2C12)	[254]
Corylifol A	10, 50 and 100 nM	muscle atrophy (↓); myoblast (↑)	Cell line (mouse C2C12)	[255]
Daidzein	50 nM	MyoD (↑), MyH (↑), myotube diameter and number (↑)	Cell line (mouse C2C12)	[257]
Sulforaphane	1 mg/kg	body weight (↑)	Animal (mouse, C57BL/6J)	[259]
Delphinidin	20 mg/kg	MuRF1 (↓)	Animal (mouse, C57BL/6J)	[260]
Isobavachalcone	100 pM	MyH protein (↑)	Cell line (mouse C2C12)	[262]
Glyoxylic acid	0.2, 0.4, and 0.8 mM	MyH II (↑), MyoD (↑), and MyoG (↑)	Cell line (mouse C2C12)	[264]
Oleocanthal	10 µM	muscle wasting (↓)	Cell line (mouse C2C12)	[265]
Panduratin A	1–40 µM	myotube diameter (↑)	Cell line (TNF-α-treated L6 SM cells)	[266]
Sophoranone	10 μM	creatine kinase levels (↑)	Animal (rabbit model of acute medial rectus muscle injury)	[267]
Diosmin	50 μM	proliferation (↑)	Cell line (mouse C2C12)	[268]
Ginsenoside Rg5	100 nM	p38MAPK phosphorylation and MyoD/E2A heterodimerization (↑)	Cell line (mouse C2C12)	[269]
Resveratrol	Diet supplemented with 0.4% (*w*/*w*) resveratrol	MSCs proliferation and differentiation (↑)	Animal (injured obese mice)	[271]
Maslinic Acid	Diet supplemented with 0.27% (*w*/*w*) maslinic acid	denervation-induced muscle atrophy (↓)	Animal (mouse gastrocnemius muscle)	[273]
Ferulic acid		MyoD (↑), MyoG (↑), and Myf5 (↑)	Zebrafish (adult male)	[274]
Theaflavin	20 μM	myogenesis (↑)	Cell line (mouse C2C12)	[275]
Paeoniflorin	50 mg/kg	weight loss and muscle fiber atrophy (↓)	Animal (C26 tumor-bearing mice)	[276]
	10, 30, and 100 μM	IL-6 production (↓)	Cell line (C26 cancer cells)	

Evidence source is standardized as Human, Animal (mouse/rat), Primary cells, or Cell line to clarify translational context.

## Data Availability

No new data were created or analyzed in this study. Data sharing is not applicable.

## Abbreviations

SM: Skeletal muscle, MSCs: Muscle stem (satellite) cells, MRFs: Myogenic regulatory factors, MyoG: Myogenin, ECM: Extracellular matrix, ROS: Reactive oxygen species, MSTN: Myostatin, T2D: Type 2 diabetes, MGP: Matrix Gla protein, MyH: Myosin heavy chain, DXM: Dexamethasone, RG: Red ginseng.
